# Building a Digital Manufacturing as a Service Ecosystem for Catena-X

**DOI:** 10.3390/s23177396

**Published:** 2023-08-24

**Authors:** Felix Schöppenthau, Florian Patzer, Boris Schnebel, Kym Watson, Nikita Baryschnikov, Birgit Obst, Yashkumar Chauhan, Domenik Kaever, Thomas Usländer, Piyush Kulkarni

**Affiliations:** 1 Fraunhofer IOSB, Fraunhoferstraße 1, 76131 Karlsruhe, Germany; florian.patzer@iosb.fraunhofer.de (F.P.); boris.schnebel@iosb-extern.fraunhofer.de (B.S.); kym.watson@iosb.fraunhofer.de (K.W.); thomas.uslaender@iosb.fraunhofer.de (T.U.); 2 ISTOS GmbH, Kaistraße 16a, 40221 Düsseldorf, Germany; nikita.baryschnikov@istos.com; 3 Siemens AG, Technology, Otto-Hahn-Ring 6, 81739 München, Germany; birgit.obst@siemens.com (B.O.); domenik.kaever@siemens.com (D.K.); 4 up2parts GmbH, Dr.-Müller Straße 26, 92637 Weiden in der Oberpfalz, Germany; yashkumar.chauhan@up2parts.com; 5 mipart GmbH, Dr.-Müller Straße 26, 92637 Weiden in der Oberpfalz, Germany; piyush.kulkarni@mipart.com

**Keywords:** Manufacturing as a Service (MaaS), digital ecosystem, production ecosystem, reference architecture, Smart Factory Web, smart manufacturing, Industry 4.0, Catena-X, Gaia-X, Manufacturing-X

## Abstract

Manufacturing as a Service (MaaS) enables a paradigm shift in the current manufacturing landscape, from integrated production and inflexible, fragile supply chains to open production and flexible, robust supply chains. As part of this evolution, new scaling effects for production capacities and customer segments are possible. This article describes how to accomplish this paradigm shift for the automotive industry by building a digital MaaS ecosystem for the large-scale automotive innovation project Catena-X, which aims at a standardized global data exchange based on European values. A digital MaaS ecosystem can not only achieve scaling effects, but also realize new business models and overcome current and future challenges in the areas of legislation, sustainability, and standardization. This article analyzes the state-of-the-art of MaaS ecosystems and describes the development of a digital MaaS ecosystem based on an updated and advanced version of the reference architecture for smart connected factories, called the Smart Factory Web. Furthermore, this article describes a demonstrator for a federated MaaS marketplace for Catena-X which leverages the full technological potential of this digital ecosystem. In conclusion, the evaluation of the implemented digital ecosystem enables the advancement of the reference architecture Smart Factory Web, which can now be used as a blueprint for open, sustainable, and resilient digital manufacturing ecosystems.

## 1. Introduction

Modern production is a continuous process of transformation towards economic, ecological, and social sustainability which is supported by various activities in national ministries, as well as in the EU and UN. The German Federal Ministry of Economics and Climate Protection describes the current digital transformation in industry as an interlinking of production with modern information technology [1] and names the following observable associated developments:

“*Rigid and firmly defined value chains are becoming flexible and dynamic. Globally interconnected value networks are emerging in digital ecosystems that enable new forms of cooperation while contributing to a climate-friendly and resource-conserving future. This results in data spaces that ensure data sovereignty, security and integrity and create the conditions for innovative products and business models*.”

[1], translated by the author.

Such an open collaborative data ecosystem is created by the Catena-X initiative (https://catena-x.net/en, accessed on 6 July 2023), which is a large-scale innovation project for the automotive industry accompanied by an association (cf. Section 2.2) dedicated to develop an ecosystem for data-driven value chains for the automotive industry. In dedicated working groups, Catena-X is structured into several use cases, such as online control and simulation, demand and capacity management, modular production and Manufacturing as a Service (MaaS). MaaS can be defined as the shared use of distributed production lines and manufacturing infrastructures to produce components or products [2]. The MaaS working group [3] of Catena-X aims at supporting sustainable, resilient, and open production networks and supply chains. For this, the working group develops digital solutions for the matchmaking between demanded and offered manufacturing capabilities, whereby the latter ranges from the production of entire products down to individual manufacturing steps. The respective topics include services supporting the request and order management, enabling the interoperability to achieve a network of networks compliant with the Gaia-X (cf. Section 2.1) data sovereignty guidelines [4]. Thus, the working group concentrates on building a digital ecosystem for MaaS.

This article presents the results of a MaaS domain analysis, including business models and requirements for a digital MaaS ecosystem applicable for ecosystems such as Catena-X. To meet these requirements and to enable the identified business models, an open prototypical architecture has been created based on the reference architecture Smart Factory Web (SFW, cf. Section 2.4) for connected smart factories. The prototypical architecture is service-based and offers MaaS-relevant IT and engineering expertise as services, enabling the development of applications and the implementation of different user journeys with low effort. This architecture supports the federation of existing manufacturer networks (e.g., on-demand manufacturing or production optimization platforms) and individual manufacturers, building a network of networks for MaaS.

Furthermore, this article describes an updated version of the Smart Factory Web (called Smart Factory Web 2.0), which now provides a blueprint for open, sustainable, and resilient digital manufacturing ecosystems. The narrative is that the development of an architecture based on the presented blueprint actually leads to a digital ecosystem supporting modern MaaS business models, e.g., federated MaaS marketplaces.

This article is structured as follows. An overview of previous work is provided in Section 2. Next, the state-of-the-art of MaaS is discussed (Section 3), followed by use cases and requirements (Section 4) faced by an MaaS ecosystem, including business models. After the description of the Smart Factory Web 2.0 (Section 5), the prototypical SFW-based digital MaaS ecosystem is presented (Section 6). The article concludes with an in-depth discussion of the observed benefits of this instantiated architecture and the Smart Factory Web for the stakeholders (Section 7) and provides an outlook for the future (Section 8).

## 2. Previous Work

### 2.1. Dataspaces and Ecosystems

Based on contributions from many industry experts and dataspaces, the project OPEN DEI [5] developed the following definition: “*A data space is defined as a decentralized infrastructure for trustworthy data sharing and exchange in data ecosystems based on commonly agreed principles*”.

In [5], the authors describe three building blocks required for a dataspace:

- A data platform for effective data sharing and exchange, as well as for the engineering and deployment of data exchange and processing capabilities.
- Data marketplaces to connect providers and consumers of data and data processing applications in the framework of business transactions.
- Data sovereignty to enable each stakeholder to control access to and usage of their data following a governance scheme with specified rules.

Dataspaces are typically dedicated to a particular sector (or vertical application, such as mobility, manufacturing, agriculture, health, or energy), where multiple stakeholders provide and exploit the available data. A data source may be used in multiple dataspaces. A dataspace is a logical, federated infrastructure based on open standards and enabling the realization and evolution of new business models. There are agreed upon operational policies and rules across a dataspace, including the onboarding of new stakeholders. A fundamental principle is to create a “level playing field” for all stakeholders.

The essential concerns of a dataspace are interoperability (of data exchange and data processing, especially regarding semantics), data sovereignty, and value creation (the benefits of joining or using the dataspace).

Dataspaces are often the enabling framework for digital twin systems. A digital twin is, in essence, a digital representation of a real-world entity: “*A digital twin is a formal digital representation of some asset, process or system that captures attributes and behaviors of that entity suitable for communication, storage, interpretation or processing within a certain context*” [6].

Digital twin systems and dataspaces form a symbiosis, since interconnected digital twins require a common dataspace to function efficiently, and digital twins can be an important business use case and motivator for the dataspace [7].

The Gaia-X European Association for Data and Cloud AISBL was founded in January 2021 with the objective of developing technical solutions and a regulatory framework for federated and secure data services in business ecosystems [4]. In the words of Ref. [5], an ecosystem can be defined as the “*[…] overall system created by the activities and connections of a set of actors and infrastructure interacting according to a common set of rules. Multiple ecosystems can exist, overlap, and collaborate […]*”. Gaia-X addresses different sectors, including mobility, energy, manufacturing, finance, agriculture, public services, and healthcare. Data sovereignty, privacy and confidentiality, security, technological neutrality, and interoperability are central to Gaia-X. It is not a European cloud solution, but provides an architecture to connect various cloud service providers, in compliance with European data protection guidelines.

### 2.2. Catena-X

Catena-X [8] is an open and collaborative data ecosystem, based on the Gaia-X principles and designed to cover the entire value chain of the automotive industry. The lighthouse project was initiated in 2020 with the motivation of ensuring resilience, innovation, and revenue opportunities for the participants of the automotive industry. The Catena-X project is supported by the funding program Kopa35c of the German Federal Ministry for Economic Affairs and Climate Action.

Currently, the association has 160 members (https://catena-x.net/fileadmin/user_upload/Vereinsdokumente/Catena-X_Mitgliederliste.pdf, accessed on 13 July 2023) from various industries that are directly or indirectly related to the automotive industry.

According to Ref. [9], the goal of Catena-X is to provide an environment in which end-to-end data chains of the entire automotive value chain can be built and used collaboratively. Interoperability and data sovereignty are the top priorities for the project’s technology landscape. Open and non-discriminatory access is also a key value, ensuring that all companies can participate, regardless of their size. Therefore, a particular focus is placed on the integration of small and medium-sized enterprises (SMEs).

The approach of the collaboration is to mutually select scalable and relevant use cases for the automotive value chain and to implement them. Standards are to be formed based on various certified technologies, from which a solution portfolio of network-based applications will emerge. Ten use cases were defined at the launch of the project, e.g., circular economy, modular production, and MaaS [3].

### 2.3. Manufacturing as a Service (MaaS)

MaaS is a paradigm shift in modern manufacturing that changes existing manufacturing landscapes by focusing on sharing networked manufacturing infrastructures to manufacture products [10]. The industry expects this new manufacturing paradigm to lead to a shift from product-based to service-based manufacturing, with the goal of fulfilling the high demands of customized products, achieving better resilience to market volatility, and attaining wide-ranging collaboration [11].

There are MaaS platforms offering services in the industrial market, often in the domains of CNC machining and 3D printing [12]. Market developments in the MaaS area are very dynamic. New manufacturing marketplaces are emerging, especially in the areas of subtractive and additive manufacturing (cf. Section 2.5).

Within the context of Catena-X, an open MaaS ecosystem is being developed for the automotive industry [13]. The objective is to offer manufacturing steps, processes, resources, and production capabilities as a service, and if possible, these should be domain independent. Industrial manufacturing service customers can thus better react to material shortages and supply problems and search for alternative suppliers and supply chains via a MaaS marketplace. Specific product work steps can be requested as part of a service. Suppliers of manufacturing services can thus win new orders and establish new business relationships, as well as better use their manufacturing capacities. An MaaS ecosystem enables networking along the value chain, risk reduction through extensive manufacturing networks/alliances, and an increase in new orders from different domains, especially for SMEs, using a one entry point promotion. Thus, compliance with data sovereignty is a key criterion in order to protect the manufacturing knowledge and business interests of the participating companies.

### 2.4. Smart Factory Web (SFW)

The Smart Factory Web is a reference architecture for open, sustainable, and resilient digital manufacturing ecosystems. The SFW was originally the architecture used for a testbed of the Industry IoT Consortium (IIC). The testbed contains a prototypical cloud platform developed by Fraunhofer IOSB and is operated by Fraunhofer IOSB, together with the Korea Electronics Technology Institute (KETI). The platform stores and offers production capabilities using open international standards such as the OPC UA and AutomationML [14]. The current reference architecture Smart Factory Web, was derived from the experience and knowledge acquired in this testbed [15]. In addition to the IIC testbed, further open specifications of Plattform Industrie 4.0, International Data Spaces (IDS), and Gaia-X have been applied to the prototype, resulting in an extension of the SFW [16,17]. Sustainability approaches, including work on digital product passports, were also investigated [18]. In Catena-X, the SFW has been applied to digitally enable various MaaS use cases and business models (cf. Section 4.1 and Section 4.3). Since the technological requirements in Catena-X partially differed from those of the IIC testbed, the prototypical implementation of the SFW was reimplemented. Moreover, from the knowledge gained in Catena-X, the Fraunhofer IOSB has refactored the SFW, resulting in a new version 2.0 (cf. Section 5).

### 2.5. On-Demand Manufacturing (ODM) Platforms

Westkämpfer (1997) defines manufacturing on demand as follows [19]: “*The opening of production networks and immediately changeable (adaptive) company structures lead to new concepts which allow an extreme customer orientation and production only by customer order, also called ‘manufacturing on demand’*”.

ODM platforms usually have a network of suppliers able to deliver the products or parts requested by the customer via the ODM platform in the desired quantity, at the requested price, and in a specified time interval. This is made possible by digitalized processes, existing partner structures, and global supplier networks [20]. In contrast to traditional manufacturing, ODM solutions provide their capacities only on demand and only for a specific period of time [21].

Table 1 provides an overview of several existing ODM platforms with their respective manufacturing domains and headquarters. This is a snapshot, as the market for ODM platforms changes continually. This list was presented for the first time in Ref. [14]. However, some platforms in the original list no longer exist, have been taken over by larger players, or have expanded their manufacturing scope. Moreover, new platforms have emerged.

## 3. State of the Art

For the development of a digital MaaS ecosystem, it is important to consider the current literature and existing approaches. Therefore, a systematic literature search was conducted using a keyword search in a number of databases (Google Scholar, ScienceDirect, Springer Link, Fraunhofer Publica). The following search query was defined for this purpose:

*(“Manufacturing as a Service” OR “Production as a Service”) AND (“blueprint architecture” OR “reference architecture”) AND “marketplace”.*

Initially, “cloud manufacturing” was utilized as a keyword as well, but this proved to add only irrelevant topics to the literature because the relevant articles were already covered by “Manufacturing as a Service” and “Production as a Service”. Thus, it was not included in the final search query.

The search in the literature databases filtered out results published before 2019. A systematic literature search (status as of 30 May 2023) yielded 11 relevant literature sources, which were assessed and described below. External literature sources and sources published by Fraunhofer IOSB on the topic of the Smart Factory Web are clustered separately.

### 3.1. Analysis of Sources Published by Fraunhofer IOSB on the Topic of the Smart Factory Web

In their article, Usländer et al. (2021) [14] describe the first version of the SFW, a reference architecture for digital marketplaces for industrial production. The SFW is based on an open technology–agnostic approach incorporating international standards. The SFW reference architecture is built to meet the functional and non-functional system requirements of platform economy business models and enables the federation of marketplaces for industrial production ecosystems.

The reference architecture Smart Factory Web, described in Section 5, is a newer version of the reference architecture proposed by Usländer et al. [14] and adapted to the Catena-X MaaS use case.

Usländer et al. (2022) [7] describe how the concept of digital twins (virtual representation of physical assets) can be combined with the concept of dataspaces. The goal is to realize digital twin applications that guarantee data sovereignty with the help of dataspaces. For this purpose, the Reference Model for Digital Twin Systems (DTS-RM) was designed, which decouples the creation and management of digital twins from their use, segmenting the virtual world into the digital twin space the dataspace and the application.

The Smart Factory Web 2.0 is compatible with DTS-RM, since it treats actors from the digital twin space and the application space, connected via a dataspace, equally (cf. Section 5.3).

Volz et al. (2021) [16] evaluates how the SFW approach can be extended with concepts of International Data Spaces (IDS) to support the secure exchange of data, using control mechanisms and data usage rules, in order to support the use cases described by Usländer and Teuscher (2022) [22]. The concept secures the exchange of critical production data between enterprises in order to build a layer of trust.

The results of this work are included in the current version of the reference architecture Smart Factory Web (cf. Section 5).

Watson et al. (2023) [18] describe the benefits of digital product passports in a network of supply chains for the development of a sustainable economy. Furthermore, a conceptual approach for the implementation and management of digital product passports within the SFW architecture is presented. In addition, two concrete use cases are described, showing how information can be derived from digital product passports and how supply chain management can be improved.

The article by Watson et al. builds on the Smart Factory Web 2.0. Therefore, this work on the digital product passport, especially the information model, is referenced in Section 6.3.

### 3.2. Analysis of External Literature Sources

Deshmukh et al. (2021) [23] describe their reference implementation of the Data Spine, which, as the authors elaborate, is “*[…] a federated platform enabler that bridges IoT interoperability gaps and enables the creation of an ecosystem of heterogeneous IoT platforms in the manufacturing domain*”. Deshmukh et al. [23] refer to the EFPF (European Factory Platform) ecosystem, which was developed as part of the European research project “EFPF: European Connected Factory Platform for Agile Manufacturing”.

According to Deshmukh et al., a federated approach based on an ontology is important so that no common shared ontology is needed, data models can be mapped, and interoperability can be improved. For the same purpose, the information and semantics concept of the SFW is instantiated with a hierarchical semantic ontology model (Section 6.3), which results in a sophisticated capability information integration concept supporting arbitrary source models for capability matchmaking. Moreover, the Data Spine focusses on information (integration) flows and is a valid candidate to cover such a workflow configuration and control for SFW implementations. However, the Data Spine is domain agnostic and therefore, does not cover the domain specifics of the SFW.

In their article, Heikkilä et al. (2022) [24] describe a service-oriented architectural approach for using robot operations as services. For this approach, robot operations are described as reconfigurable skills and the technical feasibility is shown by means of experimental tests in the field of intralogistics and robot assembly. Heikkilä et al. [24] refer to the SFW as follows: “*We share the approaches with SFW but introduce the connection between data models to robot skills and operations,* via *REST and IDS interfaces.*” The focus of Heikkilä et al. is on the approach of using robotic operations as services. In this context, the development of a digital MaaS ecosystem, building on the SFW, is of future relevance.

Gong et al. (2021) [25] provide an outlook centered on model-based approaches for crowdsourced manufacturing so that “cyber manufacturing capabilities” can be offered as a service. In this context, Gong et al. describe an MaaS use case for crowdsourced manufacturing in the field of 3D printing in orthodontics. The work of Gong et al. is a review article, and the technological aspects were not considered in detail.

Landolfi et al. (2019) [26] describe a reference architecture for an MaaS platform architecture called MANUSQUARE, which has been developed in an EU project of the same name (www.manusquare.eu, accessed on 17 July 2023). MANUSQUARE enables an ecosystem and includes a virtual marketplace that connects manufacturing service providers and consumers. It focuses on the integration of IDS connectors for secure trusted data exchange and a product life cycle assessment. The reference architecture of Landolfi et al. is similar, in some approaches, to the SFW. However, the MANUSQUARE architecture provides a gateway orchestrator, which implements the business processes for which the architecture is used. The SFW, on the other hand, does not define how the business processes are implemented and leaves the service orchestration to the service brokers (specifically, the applications implementing the business logics). Moreover, the MANUSQUARE suggests single Application Programming Interfaces (APIs) for the gateway orchestrator and an “Ecosystem Data Manager”, which is a monolithic data management and data flow application. In contrast, the SFW suggests the use of services with respective APIs to provide purpose-driven APIs and lightweight service implementations to allow more fine granular access control, better scalability, and an open, unrestricted service landscape. The MANUSQUARE architecture is highly adapted to the IDS architecture. Nevertheless, the authors seem to have misinterpreted the IDS reference architecture, which can be seen by the misuse of internal and external connectors (cf. Figure 2 in [26] as compared to [27]). Unfortunately, the software developed in the MANUSQUARE project is no longer available and can neither be used nor reviewed.

Järvenpää et al. (2018) [28] describe the ontology engineering process for the OWL-based manufacturing resource capability ontology (MaRCO) to be used for matchmaking in an MaaS system. The authors consider a family of interlinked models: the process taxonomy model, the product model, the resource model, and the capability model. A capability is described by characteristic parameters and may be a combination of capabilities. The combined capabilities of resource aggregations are inferred using SPARQL Inferencing Notation (SPIN).

In a more recent article, Järvenpää et al. (2023) [29] define a layered architecture for a capability matching system based on an ontology. The matchmaking ontology imports ontologies for resources and products. As in [28], the authors apply a rule-based approach for inferring the capabilities and parameters of resource combinations. The matching process has the objective of finding a suitable resource combination and is conducted first at the capability name level, then at the level of interfaces between resources, and finally, at the parameter (property) level, but does not consider resource availability.

To achieve semantic interoperability, a manufacturing capability ontology is a valid option for a digital MaaS ecosystem, where the standardization of all semantic terms and constraints cannot be expected. For this reason, Section 6.3 presents an ontology developed by Fraunhofer IOSB that adopts similar conceptual approaches, at the top level, as the work of [28,29], but also includes additional concepts for supply chains and digital product passports. For a future extension of the search abilities of the prototype described in Section 6.1.5, the approaches of [28,29] will be considered, especially the matchmaking system and the application of SPIN rule language.

Hasan and Starly (2020) [30] describe a cloud-based middleware for MaaS, utilizing distributed ledger technology. For this, the authors propose an object-oriented smart contract system.

The digital MaaS ecosystem in the article at hand does not consider distributed ledger technology or smart contracts for capability information management to omit redundancies and the low scalability of semantic integration and information linkage. Furthermore, dataspace connectors are used as an enabling technology for the secure and sovereign data exchange between organizations to ensure privacy, ownership, and traceability. However, for future developments regarding other types of information (e.g., for order management or supply chain sustainability), the tracking of digital asset data (e.g., using distributed ledger technology) and the conclusion of electronic contracts (e.g., smart contracts) could become important.

The literature review identified existing work in the area of MaaS ecosystems, derived relevant aspects for this work, and determined that digital MaaS ecosystems can play a significant role in future production landscapes.

## 4. Use Cases and Requirements for a Manufacturing as a Service Ecosystem

### 4.1. MaaS Use Cases

MaaS in Catena-X concentrates on the matchmaking between the manufacturing capability demand and the offerings. In this context, capability refers to an asset’s ability to produce products and their properties, or the methods used for production [14]. For this purpose, the following two use cases have been defined.

**Figure 1:**
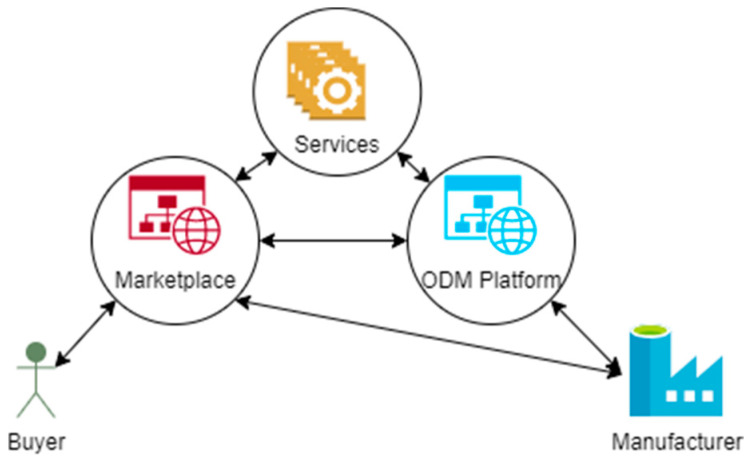
Overview of the federated marketplace use case.

The first use case builds on a **federated marketplace** for manufacturing capabilities (cf. Figure 1). For this use, manufacturers (cf. Table 2) register their capabilities directly in the federated marketplace or by onboarding on another MaaS marketplace (e.g., an ODM platform) which registers their capabilities for them as a proxy. Capabilities include knowledge about the factories, e.g., machinery, human expertise, past production outputs and processes, capability terms like “band sawing”, certifications, etc. Buyers (cf. Table 2) are further able to search for the manufacturing capabilities they require to produce a certain product (usually parts or assemblies). This can include individual manufacturing steps to cover missing capabilities in regards to existing productions or even fully external production offers. Both the ODM platform and the marketplace can perform the necessary matchmaking between required and offered capabilities. Usually, the ODM platforms offer the production of a certain part and often act as manufacturers, whereas the marketplace only offers the production by an arbitrary number of manufacturers. However, both may use additional IT or engineering services to provide their customers with added values such as feasibility analysis or optimization for cost reduction. Moreover, marketplaces can build hierarchies within themselves or with ODM platforms. This is the case in Catena-X, where a marketplace not only offers manufacturing capabilities registered directly by the manufacturers, but also capabilities registered with ODM platforms. In consequence, a network of networks is built which connects different networks of manufacturers (according to their capabilities) and offers multiple layers of monetarization and added value. This approach enables a single-entry point to the network of networks via the marketplace.

**Figure 2:**
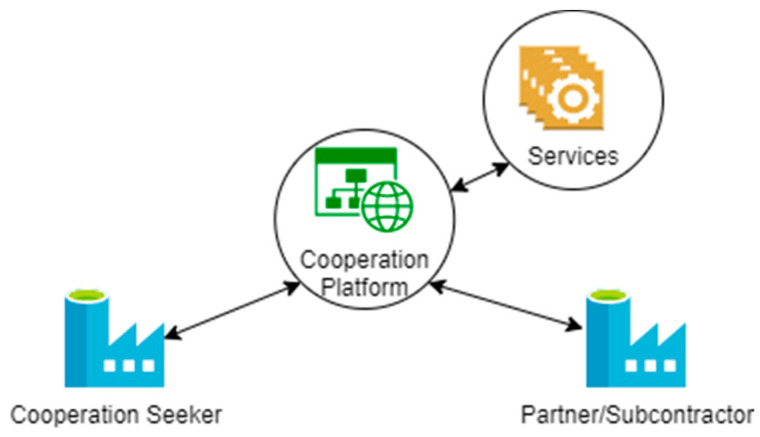
Overview of the cooperation platform use case.

Similar to the federated marketplace use case, the **cooperation platform** use case consists of platforms offering the matchmaking between required and offered manufacturing capabilities, as well as the possibility to leverage IT and engineering services (cf. Figure 2). However, this use case is motivated by the search for cooperative partners in order to fulfill customer orders. Therefore, the cooperation seeker (cf. Table 2) tries to find a partner/subcontractor (cf. Table 2) able to supplement their own manufacturing capabilities in order to fulfill a contract with a customer.

The stakeholders in Table 2 have been identified from the use cases and will be referred to in the rest of the article.

### 4.2. Business Value

The success of MaaS depends, to a large extent, on the business benefits of the stakeholders (Section 4.1). Hence, this section examines the tangible and intangible benefits of MaaS.

Buyers want to outsource certain manufacturing steps via MaaS. One reason could be that existing suppliers are currently unable to deliver, and that in-house production is currently not possible due to resource, capacity, or material bottlenecks. Traditional processes for sourcing and selecting manufacturers involve considerable effort, such as searching for and communicating with potential manufacturers, or comparing different offers. MaaS simplifies and accelerates this process by enabling buyers to multicast their requests in an IP-secured manner across a network of manufacturers and production networks and automatically match their demands with suitable suppliers and supply chains.

In addition, an MaaS platform can consider factors such as capacities, costs, processing times, energy consumption, or supplier ratings. There are MaaS platforms claiming to reduce the time needed to fulfil an order by 50%, on average [31]. With the help of the intelligent matching of requirement and manufacturing capabilities, the transaction effort and associated labor costs are significantly reduced. This effect can be further increased by using the services offered to automate workflows for automatic data preparation, costing, or contract and payment processes [32].

MaaS also serves as an open marketplace for manufacturers to showcase their capabilities and integrate their machines to increase orders and maximize utilization. Higher machine utilization has a positive impact on the total cost of ownership, since more parts can be produced during the utilization phase. As reported in [33], an analysis of a TRUMPF laser cutting machine for metal sheets, conducted by SYSTEMIQ and TRUMPF, shows that equipment as a service, which can be seen as a subset of MaaS, has the potential to reduce the total cost of ownership by approximately 16–24%, depending on the scenario. In the same analysis, MaaS concepts were found to have the potential to reduce the carbon footprint over the entire product life cycle. When the machine was offered on a marketplace platform, emissions in metal sheet processing could be reduced by up to about 65%.

After discussing the business benefits enabled by MaaS for stakeholders, the next chapter focuses on the underlying business models that are possible within MaaS.

### 4.3. Business Models

MaaS as part of Catena-X offers a range of functionalities, mechanisms, and services within a defined reference architecture. Both the entire marketplace and individual solution applications have the potential for the creation of specific business models. The simplest business model for service applications is a pay-per-use mechanism. For the use of platform functionalities, licensing models are conceivable, as are equidistant fees for operating the platform. The payment is split between the participants who generate profits at the various endpoints of the market hub.

In a previous journal article by Usländer et al. [14], 55 innovative and widely used business models, described in Ref. [34], were evaluated regarding their relevance for an MaaS ecosystem. Of these, the following are highly relevant for potential operators of a platform built on the reference architecture Smart Factory Web:

- **BM2 Affiliation**: The affiliation business model aligns offers and demands, supports other parties in marketing products, enables successful transactions, and profits from them. It is well suited to MaaS applications because it minimizes the additional sales and marketing effort on the vendor side, which is often a challenge, especially for small companies, e.g., in the automotive sector. Affiliation is particularly effective in strong ecosystems in which vendors have a clear understanding of their target customers. Monetization is usually achieved via pay-per-sales systems or order fees.
- **BM52 Two-Sided Market**: Similar to affiliation, the two-sided market model brings together two sides (buyers and providers) seeking each other. The two-sided market business model benefits significantly from network effects, becoming more attractive as the number of participants on both sides increases. Additionally, it is possible to incorporate additional target groups and create a multi-sided market, for example, by allowing digital service providers to offer their products and services in an MaaS ecosystem as well. The two-sided market model can be offered free of charge for search purposes, or it can be transaction-based or funded by participation fees.
- **BM34 Orchestrator**: The orchestrator business model is widespread in the MaaS/ODM area. In this model, the platform acts as the director of the value chain and coordinates the required value creation activities. This relieves customers from investing their time, since MaaS providers as specialists who can perform these tasks much more efficiently. They have strong networks and know the core competencies of the companies involved. In this model, the MaaS provider is often the customer’s contractual partner. Transaction costs are usually higher because the platform performs more work on behalf of the customer.
- **BM35 Pay-Per-Use**: The pay-per-use model can be combined with the other business models mentioned. In this model, the customers only pay when they actually use the products or services. This offers the customer great flexibility. Customers are not charged high fees for products or services they do not use regularly. In the context of MaaS, pay-per-use can be applied to successful transactions, search services, or other workflow-supporting services that are charged to the customer when used.
- **BM48 Subscription**: The subscription model allows customers to access products or services at any time for a regular fee. Unlike the pay-per-use model, customers do not have to pay on a transaction basis to use the platform, which saves both time and money in the case of regular use. For platform providers, the subscription model offers the advantage of predictable and recurring revenue compared to the pay-per-use model.

### 4.4. Functional and Non-Functional Requirements

Functional and non-functional requirements must be fulfilled for the development of a digital MaaS ecosystem. These requirements have an impact on the architecture of the MaaS ecosystem and its functionalities. A previous journal article by Usländer et al. [14] describes the functional and non-functional requirements for federated marketplaces. Since the article at hand focusses on the application of digital MaaS ecosystems for use cases, such as federated marketplaces, these requirements have been taken as a starting point and were extended, as depicted in Table 3 and Table 4.

## 5. Reference Architecture Smart Factory Web 2.0 (SFW)

The purpose of the reference architecture Smart Factory Web 2.0 (further written without the version number) is the provision of a blueprint for a digital ecosystem enabling various manufacturing use cases at once, while supporting modern, especially data-driven, ecosystems. For this purpose, the reference architecture supports dataspace technology and is designed to be generic enough to allow the implementation of common governance mechanisms such as the ones required by Gaia-X.

Furthermore, the SFW focusses on the following architectural design goals:

1. Capability information extraction, integration, and management without requiring full cross-domain standardization;
2. An open architectural approach to omit vendor-lock-in effects and enable extensibility;
3. Enabling of service- as well as platform-based business models;
4. Flexibility regarding security and data sovereignty mechanisms;
5. Support integration into other ecosystems, e.g., to enable MaaS use cases in use-case–agnostic ecosystem implementations;
6. Scalability and distributed operability.

The basic idea of the SFW is to provide a consistent digital infrastructure including applications and services relevant for smart connected factories, which external applications and services can use at will. This is the basis for open and highly extendable digital ecosystems. At the same time, this allows for the creation of open service and data markets.

### 5.1. System Context

**Figure 3:**
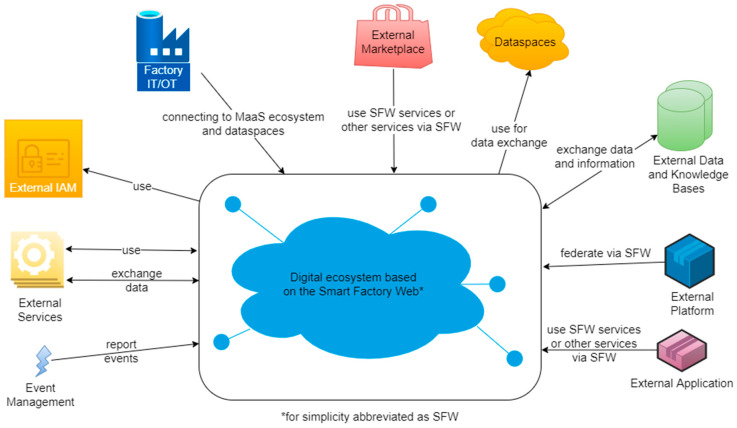
System context of the SFW to provide an overview of the necessary interfaces and interoperability requirements.

Particularly due to design goals 2–5, digital ecosystems following the SFW possess interfaces and interoperability dependencies on many external systems. Figure 3 provides examples of such systems, covering security mechanisms (cf. identity and access management (IAM)), factory components, cloud and dataspace applications, as well as external data sources and services.

These system interfaces are mostly defined by the digital ecosystem based on the SFW and its services. This includes arbitrary service interfaces, but as a minimum, APIs are required for information management and searching, since these are key aspects of an SFW-based digital ecosystem.

### 5.2. Business Logic

**Figure 4:**
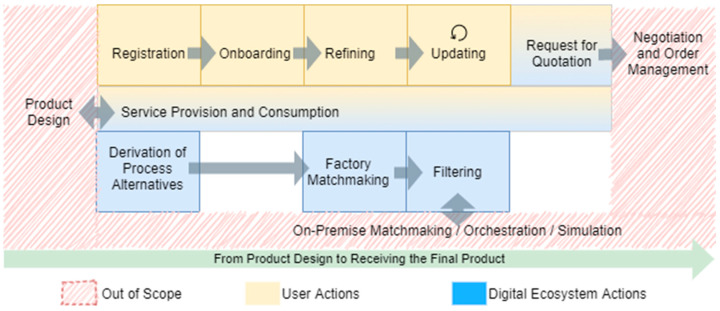
Phases of a capability matchmaking MaaS use case.

In general, the SFW does not consider specific business logic. However, the SFW is designed to support service- and platform-based MaaS business models, as described in Section 4.3, and to use cases such as those described in Section 4.1. Thus, capability matchmaking is a central requirement for every SFW-based architecture. This leads to the phases depicted in Figure 4. The figure segments the phases into phases containing ecosystem and user actions. For the matchmaking to work, manufacturers must register themselves with the ecosystem in order to onboard their capability-related information. Furthermore, since they have the domain-expertise regarding their capabilities, they refine them (cf. Section 6.1.7), which from their point of view, is similar to search engine optimization and keeping their information up to date. For these tasks, they can use services provided by the digital ecosystem (cf. service provision and consumption). Stakeholders with a demand for manufacturing capabilities can additionally use services to communicate this demand to the systems which perform the matchmaking. Moreover, if the demand is not yet available as process alternatives or similar machine-interpretable structures, it has to be transformed accordingly. If manufacturers have been identified by their matching capabilities, they must be further filtered according to additional aspects, e.g., carbon footprints, to fit respective prerequisites for an order. Thus, the information of interest must be requested from factory endpoints or additional services.

Figure 4 also depicts phases which are currently out of the scope of the SFW. This includes the product design, the simulation and planning within a factory, and all steps generated after a request for a quotation is submitted.

### 5.3. Roles

**Figure 5:**
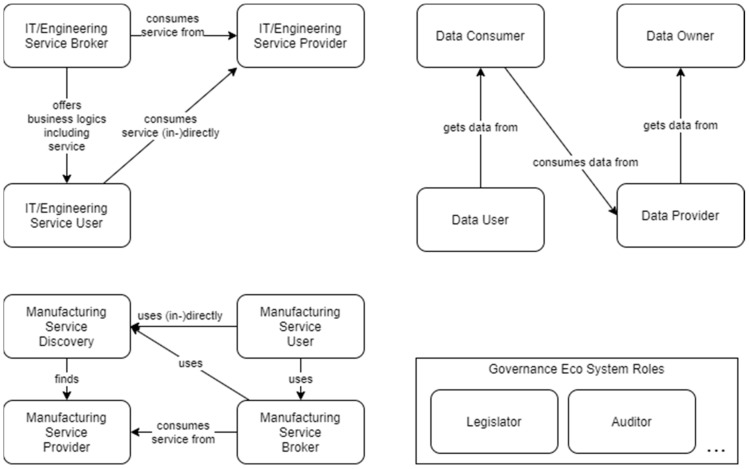
Roles within the Smart Factory Web.

An SFW-based digital ecosystem includes, but is not limited to, the roles depicted in Figure 5 and explained in Table 5. Note that a stakeholder can fulfill multiple roles, such as IT/Engineering Service Broker and Manufacturing Service Broker or Manufacturing Service User and Data User.

The SFW does not define the ecosystem enabled by the digital ecosystem; thus, it does not explicitly define ecosystem roles such as Legislator or Auditor, although they might also interact with the digital ecosystem.

**Figure 6:**
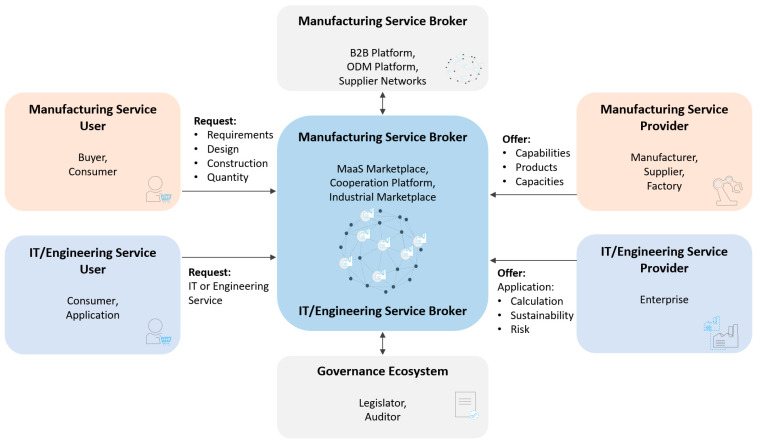
Allocated roles and examples of information exchange.

Figure 6 depicts how the roles can be mapped (assigned) to stakeholders and technology. Moreover, it shows what information they exchange (dependent on the use case). The graphic also includes the connections between several brokers, which is an example of the federation of manufacturer networks building a network of networks.

### 5.4. Functional Architecture

**Figure 7:**
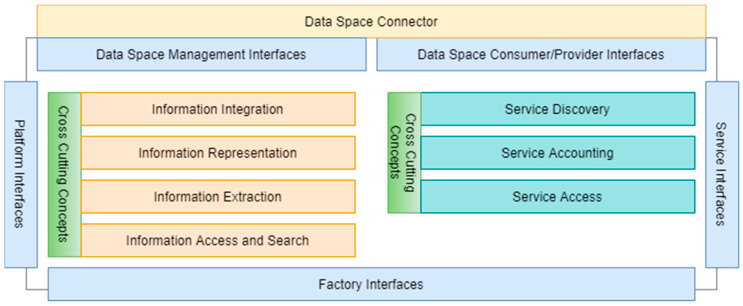
Functional overview of the SFW.

Figure 7 depicts the functional overview of the SFW. The SFW-based architecture must support five types of interfaces, including two interface types to communicate via the dataspaces, one for the management of the respective connectors, and another covering data consumer and provider interfaces, usually containing backend adapters. As the SFW builds upon services, the services’ interfaces, usually HTTP-based APIs, like REST or GraphQL, are part of the architecture as well. Moreover, the SFW does not require such interfaces to communicate via dataspace connectors, since the SFW is not restricted to dataspace usage. The same applies for the platform and factory interfaces.

As Figure 7 shows, an important functional category concentrates on information integration, representation, extraction, and access. Since the driving use cases for the SFW evolve around manufacturing capabilities and their respective matchmaking of demands and offers, this functional category is central to the SFW.

As a service-based architecture, the SFW also requires a service infrastructure for discovery, accounting, and the access to services.

The SFW does not define cross-cutting concepts such as specific security or scalability mechanisms in detail. However, they are of utmost importance in every architecture and need to be tailored to the resulting architecture and operation concept. As an example, a dataspace usually defines compliance rules involving security mechanisms which have to be implemented by the SFW-based architecture. Otherwise, its components would not be allowed to participate in the dataspace.

### 5.5. Functional Core Components

**Figure 8:**
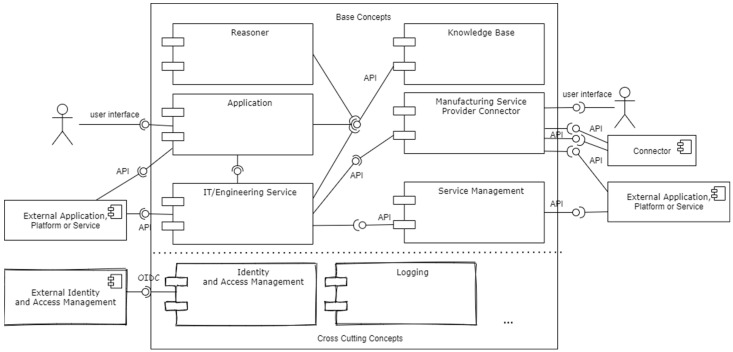
Core components of the SFW, including interfaces exposed to and offered by external components.

To implement the functions from Section 5.4, certain core components are required (cf. Figure 8). Each component can be implemented by an arbitrary number of instances.

All core components have either an API to communicate with other components or a user interface and an API client to consume APIs. IT/Engineering Service Providers can connect their external application or service to the core components via designated APIs. Manufacturing Service Brokers can connect their platforms and supplier networks. Knowledge bases and reasoners are central for information integration and therefore, are important for capability semantic integration, matchmaking, and linkage. Knowledge bases are never directly accessed by external applications. Instead, core applications and services access a knowledge base to fulfil their purposes and forward data to or from the knowledge base, while applying the necessary transformations and mappings. The manufacturing service provider connectors have the purpose of extracting relevant information, such as capabilities or the ability to meet a delivery date, from the Manufacturing Service Providers. The connector receives its information from external applications by connecting to their APIs or via another connector, in which case, each connector can expose an API, which will be consumed by the counterpart. Usually, the Manufacturing Service Provider connector must be configured by the Manufacturing Service Providers themselves, or by a consulting company, in order to achieve the necessary data transformation and mapping, unless a standardized implementation is used to provide the information (cf. Section 6.4).

Cross-cutting concept components may vary, depending on the ecosystem, but are also part of the core components.

### 5.6. Security

Besides the common security best practices, such as security by design and security by default, instantiations of the SFW are required to implement basic dataspace-related security mechanisms.

**Security Architecture**: Every SFW-based architecture is a system of systems and must include security architectures for every subsystem compliant with the dataspace/ecosystem’s overarching security architecture. As an example, knowledge bases and their management and information access services will usually be implemented within one system and be operated by one legal entity. Consequently, they must be supported by a well-defined security architecture, e.g., following the NIST framework (https://www.nist.gov/cyberframework, accessed on 6 July 2023) or the BSI standards (https://www.bsi.bund.de/EN/Themen/Unternehmen-und-Organisationen/Standards-und-Zertifizierung/IT-Grundschutz/it-grundschutz_node.html, accessed on 6 July 2023), supporting well-defined interfaces with the dataspace architecture.

**Dataspace Connectors**: As data sovereignty is a core requirement for every modern dataspace (esp. following the Gaia-X requirements), an SFW-based architecture must be able to support dataspace connectors for usage and access control. The dataspace connectors are applied when data leaves a security zone of one legal entity and enters the zone of another. This includes the data transferred over the internet, when tunneling is not applied. Additionally, connectors can be applied between security zones of one legal entity. Dataspace connectors can also be replaced by a similar technology, e.g., a Self-Sovereign Identity (SSI) alternative.

**Identity and Access Management**: All services and applications accessible via an ecosystem must support the ecosystem’s identity and access management in order to be compliant with Gaia-X. This is especially important, since rules and policies must relate to specific entities or groups of entities, and data, as well as identity sovereignty, are key aspects of many ecosystems. Therefore, an entity and its group require identities which are unique within the ecosystem, e.g., via SSI. This requirement can be partially met by the application of a dataspace connector. However, full identity management support by the dataspace connector is not necessarily the case, e.g., when a dataspace connector is acting as a transparent proxy.

### 5.7. Semantics and Data Models

Section 5.7.1 introduces the relevant information categories necessary for building MaaS ecosystems. In addition, the importance of semantic integration and linkage as supplements to common semantics is described in Section 5.7.2.

#### 5.7.1. Information Categories

In an MaaS ecosystem, data are exchanged between different stakeholders. Service providers exchange data so that buyers and suppliers can use the required functionalities of an industrial marketplace. Suppliers need to register their production capabilities so that they can be found by buyers. Buyers make specific production requests, which are interpreted and matched with suitable suppliers and supply chains. In the context of these processes, different information categories and objects are exchanged, as introduced in this section. The definitions are taken from Patzer et al. [35], unless another source is provided.

**Asset**: An Asset is a physical or logical object (Entity) owned by or under the custodial duties of an organization, having either a perceived or actual value to the organization [36]. An Asset inherits relations from an Entity.

**Entity**: An Entity is a concept which represents the commonalities of a Process, an Asset, and Capability. Each Entity may possess Properties and Semantic References.

**Property**: A Property is an attribute of an Entity which might—but does not need to possess—data.

**Semantic Reference**: A reference to an arbitrary globally uniquely identifiable semantic.

**Process**: A Process is a set of interacting operations in a system by which matter, energy, or information is transformed, transported, or stored [37]. A Process may contain sub-processes. A Process is a set of process steps or a single process step. A Process inherits relations from an Entity.

**Product**: A Product is a material good or an (immaterial) service offering which is an outcome (output product) or an input (input product) of a Process. A Product inherits relations from an Entity and an Asset.

**Product Class**: A Product Class is a group of Products which share characteristics.

**Factory**: A Factory is either a physical or a virtual production plant acting as container for Production Resources and Products. It has the child concepts Virtual Factory and Physical Factory. A Factory inherits relations from an Entity and an Asset.

**Physical Factory**: A Physical Factory is a physical production plant acting as container for Production Resources and Products. A Physical Factory inherits relations from an Entity, an Asset, and a Factory.

**Virtual Factory**: A Virtual Factory is a container for Production Resources and products which cannot be associated to a Physical Factory. A Virtual Factory inherits relations from an Entity, an Asset, and a Factory.

**Enterprise**: An Enterprise is an organization, a company, or a business containing Factories. An Enterprise inherits Properties from an Entity and an Asset.

**Production Resource**: A Production Resource is a functional unit needed to perform required operations. It differs from other resources, such as energy or raw materials. It inherits relations from an Entity and an Asset. A Production Resource is an Asset that provides the Capabilities which are required for performing a particular process step on a Product.

**Capability**: A Capability is an Entity which represents a designated function to achieve an effect in the physical or virtual world [38]. Capability inherits relations from Entity.

**Bill of Process (BoP)**: A BoP is a structure that shows different Processes and their relationships for manufacturing a Product [39]. BoP is a list of Processes required for the manufacturing of a Product.

**Bill of Materials (BoM)**: A BoM consists of the data that identifies the items or raw materials used to produce any physical thing, whether that thing is a structure or a Product [40]. It is a list of input products (including product properties) for a given manufacturing Process.

**Supply Chain**: A supply chain comprises the exchange of materials and information in the logistics process, from the procurement of raw materials to the delivery of finished Products to the end consumer [41].

**Supply Chain Element**: An Element of a Supply Chain that represents suppliers, factories, or a logistics provider.

**Product Passport**: The digital Product Passport is a key emerging technology within a digital ecosystem used to track and monitor product Properties throughout business processes in a sustainable economy (cf. [18] and the references therein).

**Human Resource**: A Human Resource is a human Production Resource that inherits all the Properties from a Production Resource.

**Machine**: A Machine is a Production Resource which represents a physical system using power to apply forces and control movement to perform an action, and it inherits all the Properties from a Production Resource.

**Location**: A Location is a container for location relevant information, such as addresses and geocoordinates. It may be referenced from an Asset.

#### 5.7.2. Semantics

Semantics provide meaning to data or information in a given context. The participating systems in an MaaS network require semantic interoperability to exchange data, with a jointly understood meaning. Semantics are important to the MaaS ecosystem because they enable the consistent interpretation of information by machines and humans. For example, they are essential for registering and searching production capabilities. The integration of MaaS applications from multiple vendors mandates commonly agreed semantics, especially to facilitate the matching of product properties to manufacturing process characteristics and to select manufacturing services. A manufacturing domain with specialized equipment has a dedicated vocabulary that often requires additional semantics to be interpreted and used correctly.

Järvenpää et al. [28] researched the knowledge representation of manufacturing capabilities. The authors state that this kind of information is based on extensive knowledge from different distributed sources in various fields of expertise, and that major issues regarding the interoperability of systems utilizing and managing this information exist. The reason given is that the majority of these systems use their own proprietary data structures and vaguely describe semantics, as well as different interpretations of the terms used and applied to describe the capabilities. According to the authors, this results in a high demand for semantic integration solutions.

Consequently, it is unlikely that common semantics will be standardized for all the different application domains in MaaS, especially considering the high level of detail necessary for capability matchmaking. Nevertheless, generic information modeling approaches exist, which provide some generic semantics, in addition to a common information structure. Two examples utilized in Catena-X are the Asset Administration Shell (AAS) [42] and the Semantic Aspect Meta Model (SAMM) (https://github.com/eclipse-esmf/esmf-semantic-aspect-meta-model, accessed on 6 July 2023). Both can be used to model the information, to some extent, and refer to other or more concrete semantics, when necessary. As an example, such semantic references can point to terms within dictionaries, like ECLASS [43] or IEC 61360 [44], covering important concepts within specific domains.

## 6. Instantiation of a Manufacturing as a Service Ecosystem

**Figure 9:**
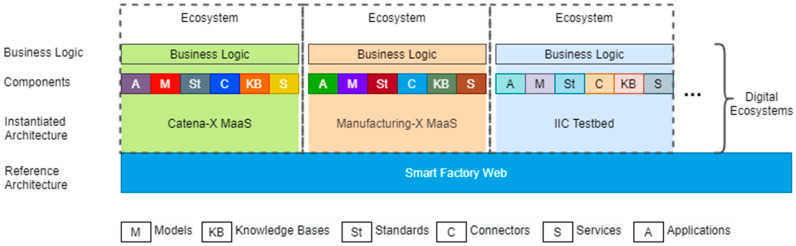
Relation of the reference architecture Smart Factory Web to ecosystems.

As Figure 9 shows, the reference architecture Smart Factory Web has been applied to build the instantiated architectures for “Catena-X MaaS”, “Manufacturing-X MaaS”, and “IIC Testbed” (in this context, an instantiated architecture is a concrete system architecture, following the generic concepts of a reference architecture). Manufacturing-X [45] is a cross-industry successor initiative to Catena-X. Here, it is representative of a concrete, yet to be approved, lighthouse project for the mechanical engineering domain. Each of these SFW instantiations represents a digital ecosystem tailored to the respective business logic and ecosystem aspects, such as governance rules, by providing its own models, knowledge bases, standards, connectors, services, and applications. As such a business logic, the federated marketplace use case (cf. Section 4.1) has been implemented on top of the Catena-X MaaS digital ecosystem, for demonstration purposes. The following section describes the pertinent implemented components.

### 6.1. Components of the Catena-X Digital Ecosystem for MaaS

**Figure 10:**
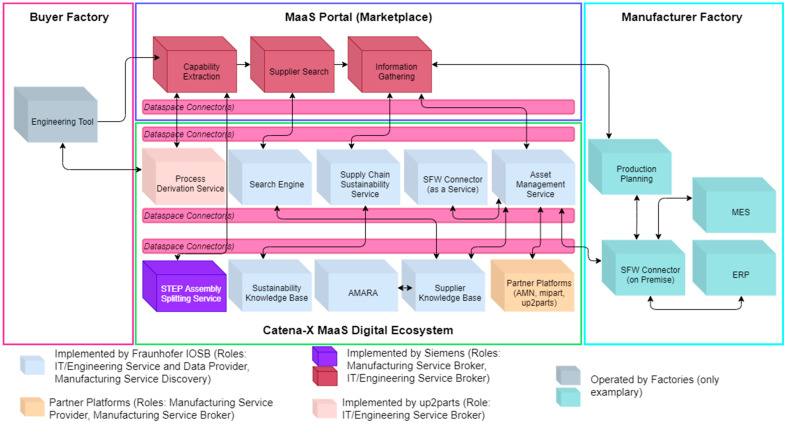
Component overview of the MaaS components in Catena-X, including their information flows.

The Catena-X MaaS digital ecosystem has been built as an SFW-based architecture, supporting the federated marketplace use case from Section 4.1. Figure 10 depicts an overview of the components and information flows developed for the Catena-X MaaS digital ecosystem, as well as components demonstrating its utilization. The engineering tool, production planning, manufacturing execution system (MES), and enterprise resource planning (ERP) components have not been implemented as demonstrators, and are only included in the overview for improved comprehensibility. The color of each component indicates the entity by which it is implemented. Additionally, the overview is segmented into the buyer and the manufacturer factories, as well as the marketplace and the digital ecosystem views. Since Catena-X applies dataspace connectors, their allocation to the respective components and information flows is also visualized in Figure 10 in a simplified manner. Here, only communication crossing enterprise borders must be secured using the dataspace connectors.

Table 6 shows which component of the instantiated architecture depicted in Figure 10 implements which SFW concept.

#### 6.1.1. MaaS Portal

**Figure 11:**
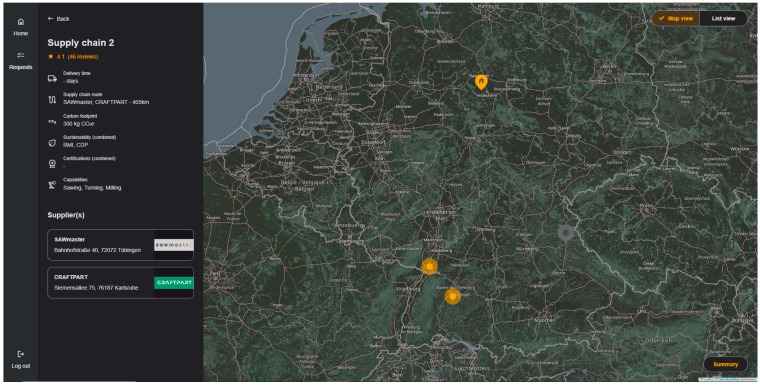
Supply chain visualization of MaaS Portal.

The MaaS Portal is the central user application for the demonstrator story. It is a cloud-based federated marketplace for connecting offers and demands within a manufacturing network of networks, as described in Section 4.1. A user account management supports the user workflow and offers a request configuration, including a user account-specific request history. After searching for potential suppliers, the graphical user interface plots a list of suppliers or shows them in a map view (cf. Figure 11), including all filter criteria information for supplier selection and quotation request. In addition, users can obtain information about the network partners and the onboarding process.

#### 6.1.2. Eclipse Dataspace Connector (EDC)

As Figure 10 depicts, the instantiated digital ecosystem leverages dataspace connectors for data sovereignty, including access and usage control. In Catena-X, the application of the Eclipse Dataspace Connector (EDC) (https://catena-x.net/en/offers/edc-the-central-component, accessed on 6 July 2023) is required to be compliant with the ecosystem’s rules. Since the EDC was still in an early phase of its development as of July 2023, the adaptation of the EDC for the components described here was still ongoing.

#### 6.1.3. Supplier Knowledge Base

This graph-based Supplier Knowledge Base [18,35] consists of supplier and capability information and is based on the supplier knowledge model described in Section 6.3. It contains and links various supplier and product information within interlinked semantically enriched knowledge graphs. Since it contains the onboarding data submitted by suppliers and ODM platforms (via the AMS, cf. Section 6.1.4), it enables the matchmaking between suppliers (not restricted to manufacturers) and buyers. To achieve this, the Supplier Knowledge Base contains several AI modules for semantic integration and automated linking to autocomplete the graphs.

#### 6.1.4. Asset Management Service

The Asset Management Service (AMS) is a core service providing a GraphQL interface to manage asset information, such as enterprises, factories, processes, production capabilities, production, and many other types of information (cf. Section 6.3), within the Supplier Knowledge Base (cf. Section 6.1.3). Among other data, suppliers and ODM platforms can register, update, and delete their manufacturing capabilities via the AMS and are thus discoverable by the Search Engine (cf. Section 6.1.5).

#### 6.1.5. Search Engine

This core service processes requirements such as production capabilities and BoM/BoP alternatives and retrieves manufacturers (more specifically, their factories) from the Supplier Knowledge Base which are able to meet these requirements. The Search Engine thus performs the matchmaking process between the supplier and the buyer, based on the onboarding information provided by these manufacturers. In the context of MaaS, the Search Engine is utilized by the MaaS Portal for searching suppliers and supply chains for a set of required production processes. For this, the Search Engine contains logic to generate SPARQL queries to navigate through the knowledge graphs in order to discover the desired capabilities. Additionally, the Search Engine calculates the coverage of the requested capabilities according to the found supplier sets.

#### 6.1.6. SFW Connector

The SFW Connector is an application (available in two options: on premise, and “as a service” via a cloud version) that enables and simplifies the exchange of information with dataspace services and applications within dataspaces. This is possible because the SFW Connector generically connects APIs with other APIs (source API to target API) and lightweight content management applications. A hybrid approach to communicate with external APIs via a combination of manual and automatic application interfaces is supported. In the future, the SFW Connector will integrate an EDC (cf. Section 6.1.2) to support communication across dataspaces. To support common organizational structures and scale to any organizational size, SFW Connectors can act as relays for other SFW Connectors and expose connected (internal) APIs.

**Figure 12:**
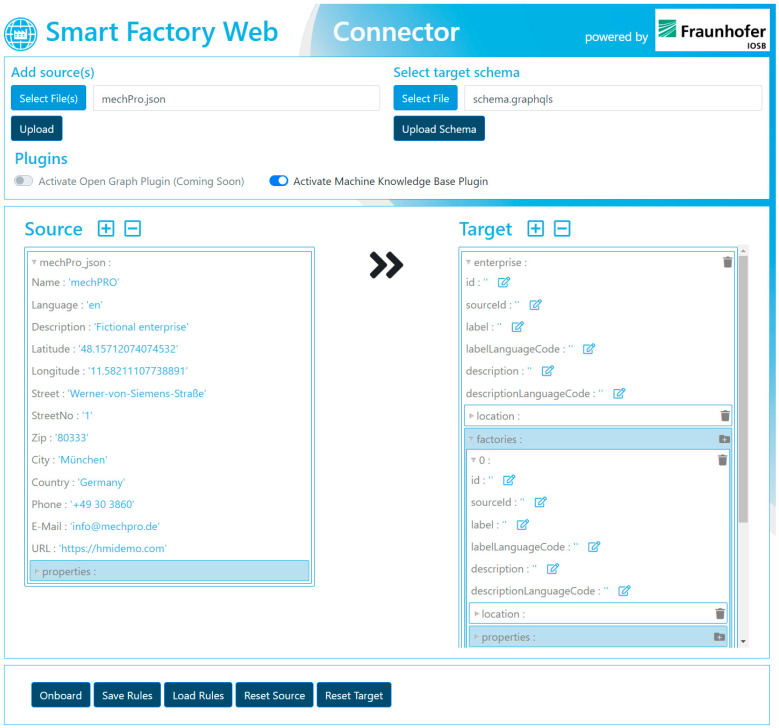
SFW Connector—data mapping from source to target.

Figure 12 shows the SFW Connector and the graphical possibility for mapping a source (e.g., a document or API) to a target source (e.g., a target schema of a database or API). In the context of MaaS, the SFW Connector is used as the application for registering the manufacturing capabilities of participating manufacturers. Companies can map different data sources, such as CSV (comma-separated values) files, AAS, and JSON (JavaScript Object Notation) to a defined target schema. The latter can be the GraphQL schema of the Asset Management Service (cf. Section 6.1.3) to onboard manufacturing capabilities, so that the SFW Connector user can be found via the Search Engine, which buyers indirectly use when searching for manufacturers via the MaaS Portal.

#### 6.1.7. Asset Management and Refinement Application (AMARA)

AMARA is the central user interface and recommender system to enable a supplier to refine its information within the Supplier Knowledge Base. Since this improves the discoverability of the suppliers, it acts as a search engine optimization tool. The semantic mapping and linking of data play a key role for a uniform interpretation of the information in the Supplier Knowledge Base (cf. Section 6.1.3). Specifically, AMARA’s recommender system supports the user in linking its own capabilities to existing ones. For example, the capability “drilling with a drill attachment for metal” can be linked to the capability “metal drilling”. AMARA allows incomplete data to be completed using several AI modules. Thus, AMARA can support suppliers with the onboarding process and the registration of production capabilities in the Supplier Knowledge Base.

#### 6.1.8. Sustainability Knowledge Base

This graph-based knowledge base integrates sustainability information from various sources and links this data from publicly available corporate sustainability reports, sustainability databases (life cycle assessment (LCA), rankings, certifications, etc.), ratings, and critiques. It is the main source for sustainability information about companies within MaaS. The graph-based Sustainability Knowledge Base is a key component of Sustainability AI (https://sustainabilityai.com, accessed on 6 July 2023), developed by Fraunhofer IOSB, and it provides transparent and comparable sustainability information regarding a wide range of industries [18,46].

#### 6.1.9. Supply Chain Sustainability Service (SCSS)

The SCSS, introduced in [18,46], enables the request of comparable, transparent, and linked sustainability information, including product carbon footprint (PCF), corporate carbon footprint (CCF), water consumption, conflict minerals, certifications, standards, and other information required for transparent sustainability assessments along supply chains [18,46]. Via a defined REST API, services and platforms can query such sustainability information on products, companies, and countries. The SCSS then retrieves the requested information from the Sustainability Knowledge Base (cf. Section 6.1.8). This also includes the required arguments and meta-information to make the results comparable. The SCSS and the Sustainability Knowledge Base are based on a uniform semantic sustainability model [46], which enables the comparability of sustainability data/assessments based on standards, calculation methods, and indicators. In the context of MaaS, the SCSS is used by the MaaS Portal to query sustainability information on the found manufacturers to supplement the result view and enable further filtering.

#### 6.1.10. Process Derivation Service (PDS)

The Process Derivation Service is primarily a concept used to extract Bills of Process (BoP) from a CAD file (in this case the STEP file format [47]) for subtractive processes like milling and turning. It is based on the up2parts cloud, consisting of various resources, materials, and raw materials. A request to the PDS must contain essential information such as a STEP file, material selection, and tolerance class. Given this information, the up2parts AI algorithm analyzes the part geometry and calculates an optimized BoP, considering factors such as the best raw material and machine resources from the master data available in an up2parts MaaS tenant. This BoP consists of multiple work plan proposals encompassing hierarchical process steps, for instance, milling or turning. Furthermore, the probabilities associated with each work plan proposal are calculated and attached, enabling the identification of the best solution. Consequently, these results are returned by the PDS as a response to the service request.

#### 6.1.11. STEP Assembly Splitting Service

**Figure 13:**
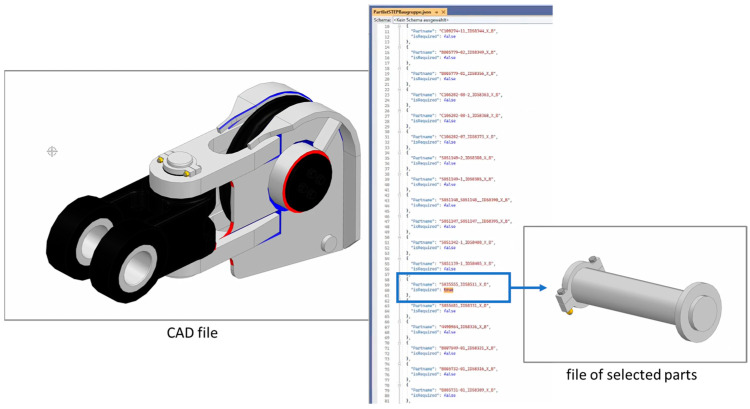
Splitting the product CAD file into component files.

The STEP Assembly Splitting Service is an offline tool that enables a user to list all included parts, mostly in tree structure, and gives the user the opportunity to select specific parts to be packed in an extra file, in STEP format (Figure 13). The remaining parts are packed in a second file. Without any selection, every part of the geometry is packed in disjunct part files in STEP format. These separated files are the output of the service. This service supports users who want to upload STEP files and if necessary, split the files easily into separate files with the configuration for different manufacturing sub-processes, such as subtractive or additive manufacturing, followed by, e.g., assembly. This service could also be used in an automated process to split assembled products into single parts to use the resulting files with the MaaS Portal.

### 6.2. Federated Marketplace—Demonstrator Stories

Leveraging the components described in Section 6.1, a demonstrator story described in this section has been implemented. The demonstrator story can be segmented into a buyer and a manufacturer story. Both stories start with the login of the user.

**Figure 14:**
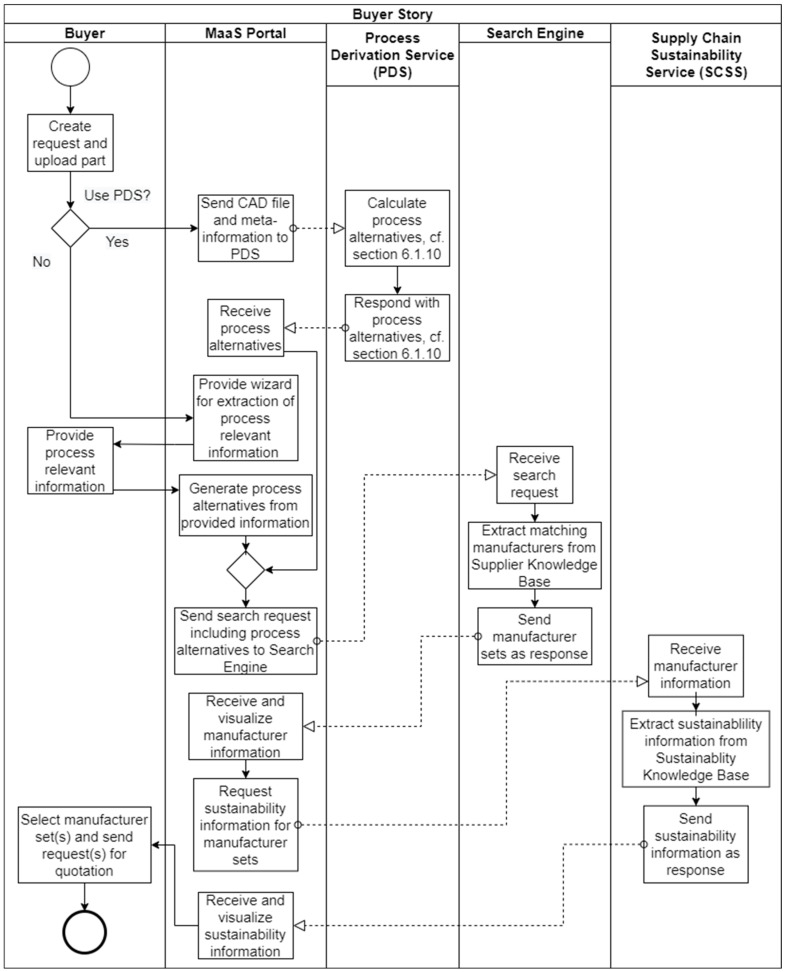
Swim lane diagram of a federated marketplace buyer story.

In the **buyer story** (cf. Figure 14), the buyer wants to search for manufacturers able to produce a certain part. The buyer is also in possession of a geometric design of this part as a CAD file (serialized into the STEP format). When the buyer accesses the federated marketplace’s website, they can start configuring a search request by providing a title, which will later identify the request in the request history. The buyer then uploads the CAD file of the part and configures, for example, the selection of the material, the required amount, or the selection of optional post-processing steps.

Before the request can be processed, the buyer selects whether the Process Derivation Service (PDS) should be used (this option only exists if the PDS covers the manufacturing domain in question, e.g., the current up2parts PDS covers subtractive manufacturing) to calculate production process alternatives for the part, or such process-relevant information will be entered manually. In the first case, the PDS will be called, given the entered request information, including the CAD file. The PDS then calculates the process alternatives, based on the up2parts AI, which has learned these from its own manufacturer network. The resulting process alternatives will then be sent to the MaaS Portal as a response. In the second case, the MaaS Portal will provide a wizard to the buyer to extract the information necessary to build process alternatives without the help of the PDS. In contrast to the PDS-based option, the buyer here needs to know how the part should be produced, without the expertise of the actual producers. However, both options will result in a set of process alternatives, which can then be sent to the Search Engine.

The Search Engine interprets the process alternatives and transforms them into SPARQL queries to extract manufacturers with matching capabilities from the Supplier Knowledge Base. Next, it returns the manufacturer information to the MaaS Portal, where it is supplemented by additional information requested from the Supply Chain Sustainability Service (SCSS), which extracts additional manufacturer-related sustainability information from the Sustainability Knowledge Base and returns it to the MaaS Portal.

The search results are displayed as supply chain visualizations. A supply chain can contain one or more manufacturers. As mentioned in Section 4.1, a manufacturer can also be an ODM platform, hiding the actual manufacturers in its network. The supply chain visualization contains information to help the buyer to select a supply chain. This includes the rating of the manufacturer, similar to the Amazon star ranking; statements about the carbon footprint of a supply chain; and information about the sustainability assessment of a company, as queried from the SCSS. Furthermore, a price can be displayed if a manufacturer can offer an instant pricing service (like the instant quoting service from up2parts). After the buyer has decided in favor of a supply chain, the request is sent to the respective manufacturer(s). In its simplest form, the request is an email sent to the manufacturer. Manufacturer networks with their own configuration interfaces receive the request via their own APIs. Moreover, at the time of the writing this article, the standardization of a request for quotation API (cf. Section 6.4.2) was ongoing, with the aim of achieving greater interoperability and scalability of this interface.

**Figure 15:**
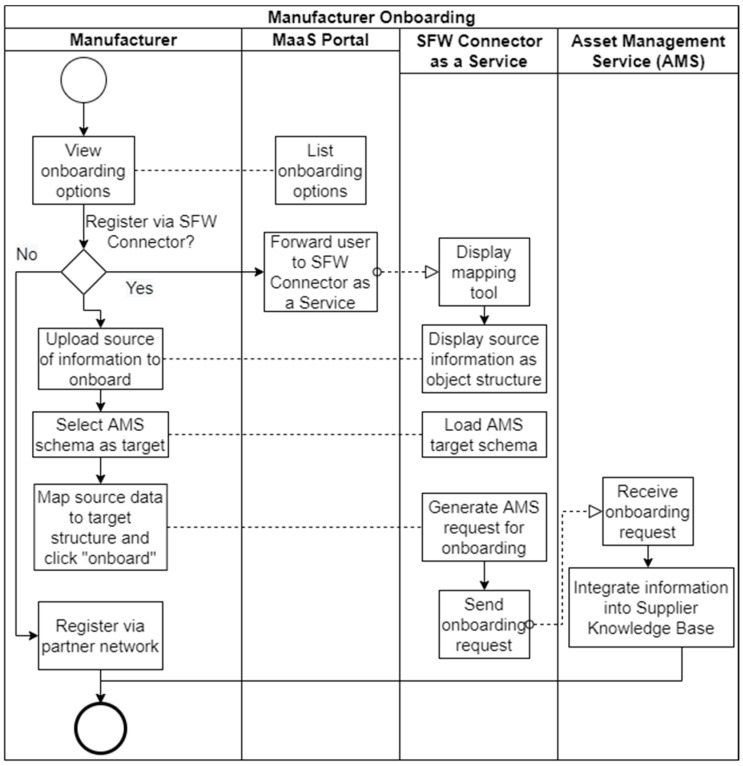
Swim lane diagram for manufacturer onboarding.

Before a new manufacturer can be considered for buyer requests, they must first onboard their capabilities into the Supplier Knowledge Base, which is covered by the **manufacturer onboarding story** (cf. Figure 15). For onboarding its capabilities, the manufacturer has two main options: onboarding via the SFW Connector or onboarding at a partner platform in order to let the platform perform the Supplier Knowledge Base onboarding for the manufacturer, utilizing the Asset Management Service (AMS). If the manufacturer uses the SFW Connector, it can access the SFW Connector directly, following a link on the MaaS Portal pointing to the cloud version of the SFW Connector (the SFW Connector as a Service). There, the manufacturer must select the AMS API schema as a target and upload its own source file or the source API schema. As described in Section 6.1.3, the user can then perform the drag-and-drop-based mapping and send the data via a one-click request to the AMS, which will integrate the information into the Supplier Knowledge Base. From there on, the manufacturer can be found via their capabilities.

### 6.3. Information Management

**Figure 16:**
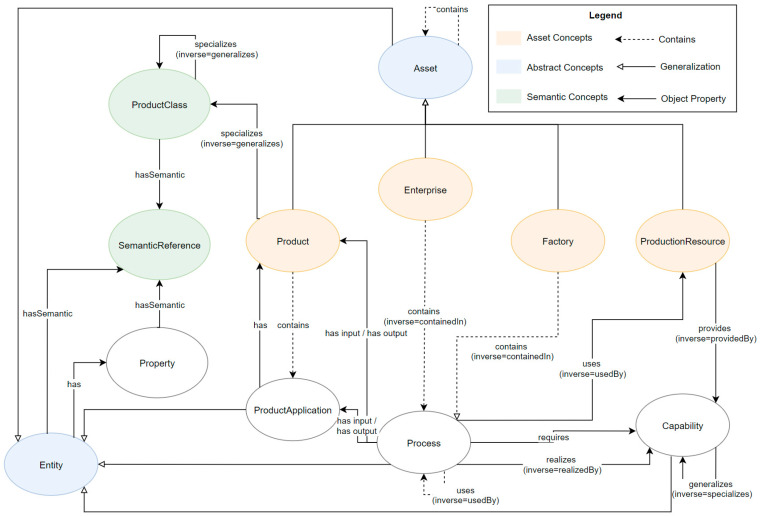
Top-level ontology adapted from Refs. [18,35].

Information management in a digital MaaS ecosystem requires a domain-independent meta model to describe the information categories, introduced in Section 5.7.1, that are addressed by the individual components for information exchange. These information categories can be mapped using a supplier knowledge model, which is used by the Supplier Knowledge Base (Section 6.1.3). The supplier knowledge model is an ontological model, as shown in Figure 16, in which some concepts are categorized as follows:

- *Asset concepts* are physical concepts in the real world.
- *Semantic concepts* provide interoperability between the physical objects.
- *Abstract concepts* encapsulate the common properties of their assigned sub-concepts.

The concepts of Property, ProductApplication, Process, and Capability are not assigned to any of these classes.

The abstract concepts Asset and Entity were introduced to encapsulate information and relationships applicable to all sub-concepts. Each enterprise can therefore contain sub-Enterprises, as well as Products, Factories, and Production Resources.

An Enterprise consists of at least one Factory, but may also contain complex structures of sub-Enterprises and sub-Factories. These sub-Factories represent the physical locations of the Enterprises. In addition, other Assets, such as Products and Production Resources, can be linked to describe the overall production structures and the product portfolio.

ProductApplications describe specific Properties, as well as quantities of an input Product, required for the respective production Process of an output Product.

The manufacturing of a Product is described with the concepts Process, Capability, and Property. A Process is realized by one or more Capabilities. Processes and Capabilities can be described more precisely by specifying their Properties.

If required, Products, Production Resources, Processes, and Capabilities can be defined additionally or explicitly at the factory level, e.g., to describe the Capabilities of a specific site in more detail.

To ensure interoperability and comparability, both within and across companies, SemanticReferences can and should be assigned to all concepts. In this way, for example, Capabilities can be mapped to each other, or Products can be compared. In addition, Products can be generalized and aggregated in classes by ProductClasses.

**Figure 17:**
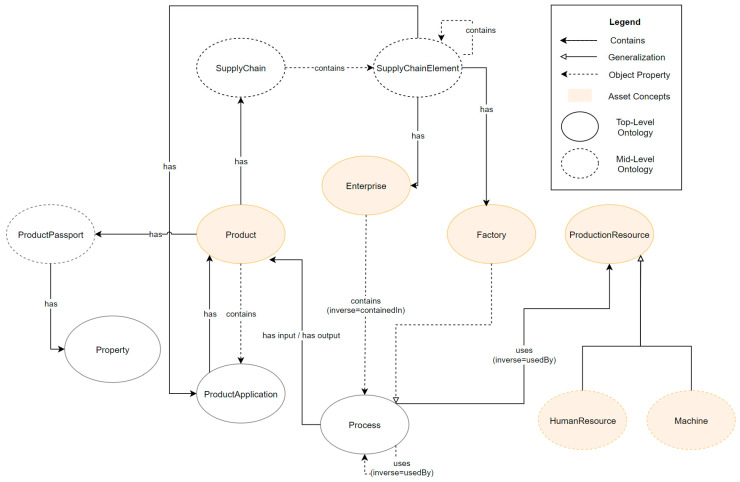
Mid-level ontology adapted from Refs. [18,35].

The following descriptions are part of the mid-level ontology (cf. Figure 17) and show the relations of these underlying concepts.

ProductionResources are subdivided into Machines and HumanResources, whereby Machines can contain anything from a production line to a sensor or small motor. Human Resources, on the other hand, describe all Assets that are represented by a human workforce.

A ProductPassport is always assigned to one Product and can contain any Properties, ranging from static physical product Properties up to dynamic lifecycle data.

A Product can have a SupplyChain. These SupplyChains contain SupplyChainElements that refer to the product suppliers, as well as to the ProductApplications of the corresponding Product.

### 6.4. Standards

Initially, the APIs and data models of the Catena-X MaaS digital ecosystem were designed and developed by the respective component developers, clustered according to the colors in Figure 10. In consequence, PDS, AMS, Search Engine, SCSS, AMN, the up2parts platform, and the mipart platform all have their own API definitions and implementations.

However, the partners identified standardization opportunities to improve interoperability of the information and communication layers. As of July 2023, two common APIs and a common data model defined by the MaaS team were in the process of becoming official Catena-X standards. The following two sections briefly describe these standardization projects.

#### 6.4.1. Manufacturing Capability Model

In a MaaS ecosystem, the components collaborate to exchange manufacturing capability information for the purpose of matchmaking. This involves the definition of resource descriptions, capability hierarchies, BoM, and BoP. Therefore, a common manufacturing capability model has been identified as a valid interoperability mechanism. As of July 2023, this data model, naturally called the Manufacturing Capability Model, was in the process of being defined and standardized, using concepts and relations similar to the ones used in the ontologies from Section 6.3. Since it is a requirement for all Catena-X data models, this model is defined in the SAMM. This data model is intended to be supported by all components of the ecosystem, transferring the information previously listed.

#### 6.4.2. APIs

Certain segments of the Manufacturing Capability Model can act as data models for common APIs. In Catena-X, two such APIs have been identified. The Request for Quotation API and the Manufacturing Capability API.

In the MaaS Portal, a request for quotation (RFQ) is created when the buyer selects a manufacturer within the results list in order to contact them for the first time. For the search, the buyer must provide relevant information such as the 3D model (STEP file), material, tolerance information, expected delivery date, quality criteria, and the technology to be used. The technology, e.g., printing (additive) or milling (subtractive), plays a vital role in evaluating the capability of the manufacturers to produce and deliver the (sub)product. All this information is not only used by the Search Engine, but is also transmitted to the selected manufacturer or the respective ODM platform as part of the RFQ. In order to reduce the efforts of integrating new ODM platforms or services, such as alternative search engines, as well as to enable interoperability between the components handling the RFQ, it was decided to standardize the RFQ interface, including the data objects mentioned previously. For some of these data objects, e.g., BoP, the Manufacturing Capability Model will be used instead of redefining them for the API.

The information represented in the Manufacturing Capability Model is transferred by a very heterogeneous set of resource databases/ERP systems, planning tools, and factory connectors. Thus, a common API improves the interoperability of these systems with each other and with the onboarding service (in this instantiation the AMS). Therefore, the API must support the provisioning and consumption of arbitrary instantiations of the Manufacturing Capability Model. As of July 2023, such an API (Manufacturing Capability API) was in the process of becoming an official Catena-X standard.

## 7. Discussion

The following discussion and validation are related to the functional and non-functional requirements (cf. Section 4.4). Based on these requirements, the degree of fulfillment of the components is evaluated (cf. Section 7.1). Section 7.2, Section 7.3, Section 7.4 and Section 7.5 mainly reflect the experience of the MaaS industry partners.

### 7.1. Requirement Fulfillment

Table 7 and Table 8 show the degree of fulfillment of the individual requirements by the instantiated digital MaaS ecosystem (cf. Section 6) and the core concepts and components required by the SFW (cf. Section 5.5).

The evaluation of the requirements has shown that the updated version of the reference architecture Smart Factory Web is suitable for building a digital MaaS ecosystem. Based on this reference architecture, it was possible to instantiate the digital ecosystem. In summary, a domain-independent metamodel has been realized which can be used for the registration of suppliers and their production capabilities. The following functionality is partially fulfilled, but can be improved: (a) search for suitable suppliers based on their capabilities and other criteria, (b) the transformation of industry-specific engineering data and the matchmaking between process description of a production request and the capabilities of registered factories, and (c) ranking, filtering, and the request for quotation. A service registry and interfaces for registering services from external providers and for connecting external platforms and supplier networks were conceptualized, but at the time of this writing, it had not yet been implemented.

### 7.2. Buyer View

Before Catena-X MaaS, buyers who needed assistance with specific manufacturing process steps or new production capabilities had to search for solutions internally, with external partners, or through domain-specific ODM platforms. In each case, buyers had to determine by themselves which manufacturing steps were required and which suppliers could be considered. This usually meant approaching multiple suppliers, manufacturers, or manufacturing platforms. Communication through diverse channels is costly and time-consuming.

A digital MaaS ecosystem, with its components (cf. Section 6.1), facilitates searching and finding manufacturers via a marketplace such as the MaaS Portal. Other benefits include access to a multi-domain network of networks, a wizard to configure and manage requests, search by part geometry, a result visualization and filter options such as supply distance between manufacturer and buyer, carbon footprint and certifications.

### 7.3. Manufacturer View

For manufacturers, in order to be found by new customers, based on their production capabilities, their production capabilities must be onboarded in the Supplier Knowledge Base (Section 6.1.3). The process is the same for ODM platforms, which can register the production capabilities of their manufacturers. Within Catena-X, production capabilities of the following platforms were registered using the SFW Connector (Section 6.1.3):

- mipart is an ODM platform that offers services such as CNC machining, 3D printing, and sheet metal processing.
- Siemens Additive Manufacturing Network is an ODM platform specializing in additive manufacturing (3D printing), as well as CNC machining, surface treatment, and finishing. It streamlines the end-to-end order-to-delivery workflow for additive manufacturing parts within organizations, factories, and with external suppliers.

In addition, production capabilities of manufacturers were registered via the up2parts cloud using AMS (Section 6.1.3):

- up2parts offers AI-based cloud software for manufacturing optimization. An interface was developed that allows up2parts customers (such as SMEs) to participate in the MaaS ecosystem as potential suppliers by onboarding their manufacturing capabilities. This integration allows SMEs to access the Catena-X network and enter the automotive sector.

Manufacturer benefits from participating in an MaaS ecosystem are as follows:

- Higher visibility (e.g., company promotion on the digital marketplace, automatic finding by new customers from arbitrary business domains, reduced sales effort).
- Increased utilization of production facilities through new orders.
- Customized production orders.
- Supporting services (e.g., instant quoting, production planning).

Moreover, the registration of production capabilities has shown that the generic SFW Connector has a high relevance in terms of data mapping from a source API to a target API.

### 7.4. Service Provider View

Service providers can offer specialized services and applications within an MaaS ecosystem, addressing areas such as manufacturing, logistics, quality control, warehousing, maintenance, and customer support.

- The PDS (Section 6.1.10) from up2parts and the STEP Assembly Splitting Service (Section 6.1.11) from Siemens are exemplary services from industrial companies participating in the MaaS ecosystem.

Advantages for service providers participating in the Catena-X MaaS ecosystem are as follows:

- Access to new industries: Participation in Catena-X MaaS as a service provider provides access to the automotive industry, a lucrative market where new collaborations and business opportunities can be developed.
- Visibility and Collaboration: Joining MaaS expands visibility and enables new collaboration opportunities, as service providers can develop services for manufacturing customers and make them available in the Catena-X ecosystem.
- Monetarization: Functionalities provided as features of a single platform can be monetarized separately and additionally via other platforms or applications.
- Efficiency and Standardization: Implementing best practices and standardization for participation in Catena-X optimizes workflows and increases operational efficiency.

### 7.5. Benefits for ODM Platform Operators

The alternative to many individual ODM platforms is to be part of one digital ecosystem. The most important advantages of participation in an ecosystem like Catena-X MaaS are listed below:

- Visibility and Acquisition: In addition to the existing user community, participation in the ecosystem allows ODM platforms to be found, not only by the ODM platform users, but also by Catena-X business partners, who use the MaaS Portal to search for suitable manufacturers. Thus, the visibility for the ODM platform operator increases with the evolution of the ecosystem. In this way, ODM platforms learn more about customer requirements and increase the number of matchmaking orders. Thus, they improve the business success of their registered manufacturers.
- Innovation and Standardization: One of the main benefits of participating in a digital ecosystem like Catena-X MaaS is the opportunity to contribute to new standards, e.g., data interface development. Another opportunity is to compare the business models of other ODM platforms in order to further develop a personal business model and adapt to the ecosystem.
- Image and Perception: Cooperation plays an increasingly important role in our economy. An ODM platform that participates in an ecosystem is more likely to be perceived as an innovative partner than a platform that conducts business in isolation from competition.
- Community and Collaboration: As part of the Catena-X MaaS ecosystem, ODM platforms can share and learn from each other. This helps them to develop faster and remain competitive. The ecosystem can also provide an opportunity for ODM platforms to offer their digital services to other ODM platforms (cf. Section 7.4), which become aware of digital service offerings through collaboration within this network of networks. The co-creation of shared services is also feasible and could be valuable.

## 8. Outlook

This section explores possible future steps and developments that can build on the current results in future projects.

In the prototypes described, the matching of buyers and manufacturers is based only on the required and offered production capabilities and properties. It is therefore recommended to extend this matchmaking using the required and provided capacities, or even the material stock, so that it can be more precise. However, matchmaking considering capacities must be accompanied by an iterative negotiation and a workflow leading to an agreement (contract) between the manufacturer and buyer, which is also included in future work. An essential enabler for this would be the automated provision of static and dynamic transaction data by manufacturers, with the help of a connection to existing systems, such as ERP systems. The result would be better support for buyers in the decision-making process, due to more precise search and matching results. This saves time in assessing quotations and order management. Manufacturers and ODM platforms also benefit from more accurate matchmaking, as requests are more precisely tailored to their specialization and capacities, which can lead to an increase in orders.

In addition to matchmaking, the process of offering, ordering, execution, and delivery is not yet considered. Including at least the monitoring of the status of a request could help to improve the bidding and execution process, as well as help to assess the quality of the delivered manufacturing services by selected manufacturers.

In addition, MaaS in Catena-X has largely dealt with the horizontal connection of different companies at the supply chain level. In future projects, it will also be important to promote vertical connectivity within companies at the shopfloor level. In order for small and medium-sized companies to remain internationally competitive, it is necessary to automate as many processes as possible, from the request for quotation to the shipment of the finished goods, or at least to support staff with digital tools. Moreover, processes such as cost calculation and quotation preparation must therefore be standardized and automated. Standardized interfaces should also enable the required data to be exchanged in the system landscape in an interoperable manner. At the shopfloor level, use cases and requirements must be developed for the automated supply of raw materials, operating resources, and software tools. This is the only way to achieve highly automated production.

Catena-X has shown that the MaaS use case attracts great interest from small and medium-sized companies wanting to participate. Therefore, the implementation of MaaS applications, standards, or interfaces by SMEs and ODM platforms in the industry on a broad basis is an important goal for empirically validating the results and for driving further developments based on their feedback and requirements.

## 9. Conclusions

This article presents a comprehensive analysis of the MaaS domain and the requirements for building architectures for digital MaaS ecosystems, enabling modern business models such as cross-domain on-demand manufacturing. The main contribution of this article is the updated version of the open reference architecture Smart Factory Web, which is a blueprint for developing digital MaaS ecosystems for embedding in ecosystems such as Catena-X. The design decisions for this reference architecture originated in a prototypical implementation of a digital MaaS ecosystem for Catena-X, as described in this article. Based on this implementation, a federated MaaS marketplace was constructed, thus demonstrating the utilization of the digital ecosystem. The demonstration showed how information integration and logic provided by a variety of services can be leveraged to quickly build MaaS applications for specific user journeys. Moreover, the prototypes demonstrated how an open network of networks can work by federating different existing manufacturer networks (e.g., ODM platforms), as well as single manufacturers, to create one network of manufacturers and their capabilities.

In the future, the implementation of the digital MaaS ecosystem will be applied to other domains and extended to cover more phases of the MaaS user journey. Based on this, the Smart Factory Web will be further tested and extended. In future work, existing products and solutions can be integrated, adapted, and extended towards interoperability within the network of networks. Additionally, the prototypes contain software which has a high potential to be further developed towards products impacting the market, such as the Process Derivation Service, the Search Engine (and its Supplier Knowledge Base, including assisting software), the SFW Connector and the MaaS Portal. This potential will be further evaluated and discussed with potential operating companies.

## Figures and Tables

**Table 1:** ODM platform examples and references.

Platform/ Company	URL *	Headquarters	Manufacturing Domain
All3DP	all3dp.com	Germany	additive manufacturing including 3D printing
beamler	beamler.com	The Netherlands	additive manufacturing including 3D printing
facturee	facturee.de	Germany	CNC machining, sheet metal processing, additive manufacturing including 3D printing, surface treatment
fictiv	fictiv.com	United States	CNC machining, injection molding, 3D printing, urethane casting, post-processing
Fractory	fractory.com	United Kingdom	sheet metal laser cutting, tube laser cutting, metal bending, CNC machining, metal surface treatment
Haizol	haizol.com	China	CNC machining, molding, fabrication, casting, stamping, post treatment
HUBS	hubs.com	The Netherlands	CNC machining, sheet metal fabrication, additive manufacturing including 3D printing, injection molding, post-processing
InstaWerk	instawerk.de	Germany	CNC machining, eroding, post-processing
LASERHUB	laserhub.com	Germany	CNC machining, laser cutting, laser cutting of tubes, bending and folding, post-processing, surface treatment
MACROFAB	macrofab.com	United States	PCBA (printed circuit board assembly)
mipart	mipart.com	Germany	CNC machining, 3D printing, sheet metal processing
PROTOLABS	protolabs.com	United States	3D printing, CNC machining, sheet metal fabrication, injection molding
Rapid Direct	rapiddirect.com	China	CNC machining, 3D printing, sheet metal fabrication, die casting, vacuum casting, injection molding, post-processing
sculpteo	sculpteo.com	France	laser cutting, additive manufacturing including 3D printing
Siemens Additive Manufacturing Network	additive-manufacturing-network.sws.siemens.com	Israel	additive manufacturing including 3D printing, CNC machining, surface treatment, finishing
spanflug	spanflug.de	Germany	CNC machining, post-processing
trinckle	trinckle.com	Germany	additive manufacturing including 3D printing, 3D customization solutions
Weerg	weerg.com	Italy	CNC machining, 3D printing, post-processing
Xometry	xometry.com	United States	additive manufacturing including 3D printing, CNC machining, sheet and tube fabrication, plastic and metal part production, value-added solutions
3YOURMIND	3yourmind.com	Germany	additive manufacturing including 3D printing, CNC machining, water jetting technologies

* accessed on 22 June 2023.

**Table 2:** Use case stakeholders.

Buyer	The buyer is a stakeholder who wants to order a component (part or assembly) to be produced externally.
Manufacturer	The manufacturer can be a single manufacturer or a manufacturing network. In the simplest case, a single manufacturer is a company with one production machine (e.g., 3D printer, lathe, etc.). A manufacturing network consists of several manufacturers that offer their manufacturing capabilities, e.g., via an ODM platform.
Platform Operator	Platform operators offer a software, usually cloud-based, which implements on-demand manufacturing, marketplace, and other use cases, including the respective user journey.
Digital Service Provider	Digital service providers offer IT or engineering logic and expertise as a digital service. They can offer their digital services to support the automation of digital manufacturing processes and reach a wider audience, thus scaling-up their business. Moreover, they support service customers in increasing their businesses.
Cooperation Seeker	Cooperation seekers are companies looking for suitable partners/subcontractors.
Partner/ Subcontractor	Partners/subcontractors are companies open to cooperation with other companies.

**Table 3:** Functional requirements (mandatory: M; optional: O) extended from the research in [14]. The stakeholders correspond those in to Table 2.

No.	Requirement	Stakeholder	Priority
FR01	Provide a domain-independent meta model to describe assets, production capabilities, properties, etc.	Platform Operator	O
FR02	User registration and authentication.	Buyer, Cooperation Seeker, Manufacturer, Service Provider	M
FR03	Onboarding of suppliers based on the meta model and open standards to describe the production capabilities and capacities of the factories.	Manufacturer	M
FR04	Search suitable suppliers based on their capabilities, capacities, prices, quality. and other criteria.	Buyer, Cooperation Seeker, Platform Operator	M
FR05	Transformation of industry-specific engineering data for the matchmaking process.	Service Provider, Buyer	M
FR06	Matchmaking between process description from a production request and the capabilities and capacities of registered factories.	Platform Operator, Service Provider	M
FR07	Ranking of search results according to user preferences.	Platform Operator, Service Provider	M
FR08	Filtering of search results by user criteria such as availability, certificates, sustainability information, quality standards, etc.	Platform Operator, Service Provider	O
FR09	Create requests for quotations. including requirements such as quantity, specifications, delivery date, price, etc.	Buyer, Cooperation Seeker	M
FR10	Provide a service registry to manage external services.	Platform Operator	O
FR11	Provide an interface (ideally open, standardized) to register services from external providers.	Platform Operator	M
FR12	Provide interfaces (ideally open, standardized) to connect external platforms and supplier networks (federations).	Platform Operator	M

**Table 4:** Non-functional requirements (mandatory: M; Optional: O) as additions to NFR01-NFR08 from Ref. [14].

No.	Requirement	Priority
NFR09	Reliability and stability of network participants and services should be high.	M
NFR10	Compliance with relevant regulations and industry standards, ensuring data privacy, intellectual property rights, and ethical manufacturing practices.	M

**Table 5:** Description of the SFW roles.

Role	Description
IT/Engineering Service Broker	Application which consumes IT or engineering services in order to cover user journeys, offering business logics and workflows for IT/Engineering Service Users.
IT/Engineering Service User	Person or application using IT or engineering services directly or through the IT/Engineering Service Broker, in order to fulfil business needs, e.g., to support engineering with feasibility analysis or costs forecast.
IT/Engineering Service Provider	Party providing IT or engineering services, e.g., feasibility analysis or costs forecast for a designed part.
Manufacturing Service User	Person (or organization) using a manufacturing service, e.g., the milling and drilling to produce a wheel suspension of their own design.
Manufacturing Service Provider	Organization providing a manufacturing service, such as milling or drilling.
Manufacturing Service Discovery	Application able to identify Manufacturing Service Providers based on manufacturing capabilities required by a Manufacturing Service User.
Manufacturing Service Broker	Application or organization providing Manufacturing Service Users with manufacturing services provided by manufacturing Service Providers. A Manufacturing Service Broker is also a Manufacturing Service Provider, if it also offers its own manufacturing services as well.

**Table 6:** Mapping of the prototype/demonstrator components to the SFW functions (cf. Section 5.4).

Component	Functions
MaaS Portal	External MaaS Marketplace
SFW Connector	Information Extraction, Factory Interface, Platform Interface
Asset Management Service	Information Extraction, Information Access, Information Integration, IT Service (including access and interface)
Supplier Knowledge Base	Information Integration, Information Representation
Search Engine	Information Access and Search, IT Service (including access and interface)
Asset Management and Refinement Application (AMARA)	Information Integration, Information Access
Supply Chain Sustainability Service	Information Access, IT Service (including access and interface)
Sustainability Knowledge Base	Information Integration, Information Representation
Process Derivation Service	Engineering Service (including access and interface)
STEP Assembly Splitting Service	Engineering Service (including access and interface)

**Table 7:** Fulfillment of functional requirements.

No.	Requirement	Fulfillment *	Components
FR01	Provide a domain-independent meta model to describe assets, production capabilities, properties, etc.	+++	Supplier Knowledge Model (Section 6.3); Manufacturing Capability Model (Section 6.4.1)
FR02	User registration and authentication.	p	Cross-Cutting Concepts (Section 5.5); MaaS Portal (Section 6.1.1)
FR03	Onboarding of suppliers based on the meta model and open standards to describe the production capabilities and capacities of the factories.	++	SFW Connector (Section 6.1.3); AMS (Section 6.1.3); Supplier Knowledge Base (Section 6.1.3); Manufacturing Capability Model/API (Section 6.4)
FR04	Search suitable suppliers based on their capabilities, capacities, prices, quality, and other criteria.	+	Search Engine (Section 6.1.5); MaaS Portal (Section 6.1.1)
FR05	Transformation of industry-specific engineering data for the matchmaking process.	++/+	Process Derivation Service (Section 6.1.10); STEP Assembly Splitting Service (Section 6.1.11)
FR06	Matchmaking between process description from a production request and the capabilities and capacities of registered factories.	+	Search Engine (Section 6.1.5); Supplier Knowledge Base (Section 6.1.3)
FR07	Ranking of search results according to user preferences.	+	MaaS Portal (Section 6.1.1)
FR08	Filtering of search results by user criteria, such as availability, certificates, sustainability information, quality standards, etc.	+	MaaS Portal (Section 6.1.1); Search Engine (Section 6.1.5); SCSS (Section 6.1.9); Sustainability Knowledge Base (Section 6.1.8)
FR09	Create requests for quotation, including requirements such as quantity, specifications, delivery date, price, etc.	+	Request for Quotation API (Section 6.4.2)
FR10	Provide service registry to manage external services.	p	Core Component (Section 5.5)
FR11	Provide an interface (ideally open and standardized) to register services from external providers.	p	Core Component (Section 5.5)
FR12	Provide interfaces (ideally open and standardized) to connect external platforms and supplier networks (federations).	p	Core Component (Section 5.5); Manufacturing Capability API (Section 6.4.2); Request for Quotation API (Section 6.4.2)

* Legend: completely fulfilled: +++; mainly fulfilled: ++; partially fulfilled, with limitations: +; possible and in conceptual planning: p; possible, but out of scope: o.

**Table 8:** Fulfillment of non-functional requirements.

No.	Requirement	Fulfillment *	Components
NFR01	Interoperability with other external platforms and supplier networks (support for federations).	+	SFW Connector (Section 6.1.3); Manufacturing Capability Model/API (Section 6.4); Request for Quotation API (Section 6.4.2)
NFR02	Applicability to new industrial domains and sub-domains.	++/+	Supplier Knowledge Model (Section 6.3); SFW Connector (Section 6.1.3); AMARA (Section 6.1.7)
NFR03	Openness in the sense of supporting international and national standards to avoid vendor lock-in and to follow future technological trends.	++/+	Supplier Knowledge Model (Section 6.3); SFW Connector (Section 6.1.3); Sustainability Knowledge Base (Section 6.1.8); Supplier Knowledge Base (Section 6.1.3)
NFR04	Adaptability to new business models.	+	Service-Based approach (Functional Architecture Section 5.4)
NFR05	Scalability of user numbers, data, platform, and service management.	+	Service-Based approach (Functional Architecture Section 5.4)
NFR06	Security to protect company data from unauthorized access and assure its integrity.	+	Security Architectures (Section 5.6); EDC (Section 6.1.2)
NFR07	Data sovereignty to maintain control over the complete processing chain of data and also the independent decision as to who is permitted to have access to it.	p	EDC (Section 6.1.2)
NFR08	Extensibility to support new types of production assets.	++	Supplier Knowledge Model (Section 6.3)
NFR09	Reliability and stability of network participants and services should be high.	o	
NFR10	Compliance with relevant regulations and industry standards, ensuring data privacy, intellectual property rights, and ethical manufacturing practices.	+	Architectural Design; SCSS (Section 6.1.9)

* Legend: mainly fulfilled: ++; partially fulfilled, with limitations: +; possible and in conceptual planning: p; possible, but out of scope: o.

## Data Availability

Not applicable.
